# The Endoplasmic Reticulum Chaperone GRP170: From Immunobiology to Cancer Therapeutics

**DOI:** 10.3389/fonc.2014.00377

**Published:** 2015-01-12

**Authors:** Hongxia Wang, Abdul Mohammad Pezeshki, Xiaofei Yu, Chunqing Guo, John R. Subjeck, Xiang-Yang Wang

**Affiliations:** ^1^Department of Human Molecular Genetics, Virginia Commonwealth University, Richmond, VA, USA; ^2^Massey Cancer Center, Virginia Commonwealth University, Richmond, VA, USA; ^3^Department of Cell Stress Biology, Roswell Park Cancer Institute, Buffalo, NY, USA; ^4^Institute of Molecular Medicine, Virginia Commonwealth University, Richmond, VA, USA

**Keywords:** endoplasmic reticulum, glucose-regulated protein 170, molecular chaperone, anti-tumor immunity, cancer vaccine

## Abstract

Glucose-regulated protein 170 (GRP170) is the largest member of glucose-regulated protein family that resides in the endoplasmic reticulum (ER). As a component of the ER chaperone network, GRP170 assists in protein folding, assembly, and transportation of secretory or transmembrane proteins. The well documented cytoprotective activity of intracellular GRP170 due to its intrinsic chaperoning property has been shown to provide a survival benefit in cancer cells during tumor progression or metastasis. Accumulating evidence shows that extracellular GRP170 displays a superior capacity in delivering tumor antigens to specialized antigen-presenting cells for cross-presentation, resulting in generation of an anti-tumor immune response dependent on cytotoxic CD8^+^ T cells. This unique feature of GRP170 provides a molecular basis for using GRP170 as an immunostimulatory adjuvant to develop a recombinant vaccine for therapeutic immunization against cancers. This review summarizes the latest findings in understanding the biological effects of GRP170 on cell functions and tumor progression. The immunomodulating activities of GRP170 during interactions with the innate and adaptive arms of the immune system as well as its therapeutic applications in cancer immunotherapy will be discussed.

## Introduction

The endoplasmic reticulum (ER) is the key organelle that plays a critical role in many cellular processes, including protein synthesis, post-translational modification, and proper folding (1). Molecular chaperones in the ER lumen, through non-covalent interactions with their client proteins, catalyze or regulate protein folding, complex formation, protein translocation, or degradation (2–6). The physiological and pathological stress conditions that disturb the highly oxidizing and calcium-rich ER environment can trigger unfolded protein response (UPR) (7, 8). The UPR is a protein quality control mechanism that aims to limit ER stress and restore ER homeostasis, in part by inducing the elevation of ER chaperones, which enhance the protein folding/refolding capacity of the ER and target misfolded proteins to the ER-associated degradation (ERAD) pathway for degradation (9, 10).

Glucose-regulated proteins (GRPs) are among the most abundant and well-characterized ER chaperones (10). As stress-inducible chaperones, GRPs were originally discovered in mammalian cells undergoing glucose deprivation (11–13). GRPs are functionally and structurally related to the heat shock proteins (HSPs) and belong to the HSP family (14–16). Unlike most of the HSPs that reside mainly in the cytosol and the nucleus, the GRPs are predominantly in the ER (15, 16). Major members of the GRP family include GRP78/Bip, GRP94/Gp96, and GRP170 [also known as oxygen-regulated protein (ORP) 150 and HYOU1] (15, 16). These GRPs are often induced by stressors that perturb the ER functions, e.g., hypoxia, nutrient deprivation, reducing agents, calcium depletion, low PH, hyper-proliferation, or viral infection (16, 17). Due to their cytoprotective and pro-survival activities (18–21), GRPs have been extensively studied in the context of cancer development and progression, including cellular signaling, proliferation, apoptosis, angiogenesis, metastasis, and resistance to therapeutics (10, 15, 22, 23). To target the autonomous tumor-promoting effect of intracellular GRPs, many approaches or agents are being developed and tested for their anticancer efficacy in the experimental models and in the clinic (15). Over the last two decades, a wealth of studies has demonstrated novel aspects of GRP functions, intracellularly or extracelluarly, in regulating innate and adaptive immune responses during interactions with the host immune system. This has provided new opportunities to develop immune-based strategies for cancer treatment (16, 24–26). As the largest GRP and molecular chaperone in the ER, GRP170 has been less studied compared to other members in the same class. In this review, we highlight recent progress in chaperoning-based diverse activity of GRP170, and discuss the potential applications of exploiting the immunological features of this molecule to design novel anticancer therapeutics.

## Grp170 and its Chaperoning Property

Grp170 was initially found in the early 1980s during a study of GRP induction by glucose starvation (13). More than 10 years later, we cloned the cDNA of mammalian GRP170 from Chinese Hamster Ovary cells (27). GRP170 has also been referred to as ORP 150 (28), which indeed is the unglycosylated form of GRP170 (28). Sequence analysis indicated that GRP170 represents a new stress protein family that is distantly related to, but different from, both HSP70 and HSP110 families (27). The HSP70, HSP110, and GRP170 families have been classified into the “HSP70 Super-Family” (14, 29). GRP170 consists of 999 amino acids, encoded by hypoxia up-regulated 1 gene (*Hyou1*) that is located on the q arm of chromosome 11. Beside glucose starvation as a classical inducer of GRPs, including GRP170, other stressors, such as hypoxia, ischemia, perturbation of calcium homeostasis, proteasome inhibitors, and non-steroidal anti-inflammatory drugs (e.g., celecoxib) are also known to upregulate GRP170 expression (13, 30–36).

Predicted secondary structural modeling indicated that the overall organization of GRP170 is similar to that of HSP70 and HSP110, but with very little similarity in C-terminal regions (14, 27, 29, 37). GRP170 also has a high degree of homology to GRP78, the ER homolog of HSP70, as they all possess an N-terminal nucleotide binding domain (NBD) followed by a β-sheet domain, which acts as the substrate binding domain (SBD) and an α-helical domain at the C terminus. The increased size of GRP170 is due to the insertion of an acidic loop in their β-sheet domain and an extended C terminus following the α-helical domain (14, 38). It has long been known that GRP170 associates with the other major GRPs (e.g., GRP78) in the ER and interacts with immunoglobulin chains (39, 40), implicating its role in protein folding or assembly in concert with other GRP or ER chaperones. GRP170 was shown to be the most efficient ATP-binding protein in microsomal extract, and was suggested to assist the translocation of polypeptides into the ER via the transporter associated with antigen processing (TAP) (41, 42). The yeast counterpart of GRP170 (Lhs1; lumenal HSP70) displayed similar activity in transporting proteins into the ER (29, 43, 44). Biochemical studies demonstrate that GRP170 is significantly more effective in blocking heat-induced protein aggregation than other stress proteins or chaperones (37, 45–48), underscoring a superior chaperoning capacity of this only ER member of the large HSP70 family.

Protein folding mediated by the GRP78-centered chaperoning machinery in the ER is regulated by its bound nucleotide, i.e., cycling of ATP and ADP (49). ADP-bound GRP78 has a high affinity for unfolded or incompletely folded proteins, while exchange of ADP for ATP decreases the affinity of GRP78 for substrates lead to release of folded protein substrate (49, 50). Surprisingly, Grp170 was recently reported to function as a nucleotide exchange factor (NEF) for GRP78 in the ER (50–52), which also raised the question of GRP170 being an independent chaperone molecule. A recent study confirmed that Grp170 can directly bind to a variety of incompletely folded protein substrates *in vivo*, although the regulation of its substrate binding function is different than for conventional HSP70 (53). GRP170 and GRP78 can associate with similar molecular forms of two substrate proteins. However, while GRP78 is released from unfolded substrates in the presence of ATP, GRP170 remains bound (53), suggesting that binding of same substrate to different GRPs may result in distinct fates for their client proteins. These data further established the GRP170 as a bona fide chaperone. More studies are necessary to address the question as to why GRP170 in the ER possess the dual ability to bind to substrates or client proteins and to have NEF activity (54).

## ER Stress and GRP170-Conferred Cytoprotection

In addition to playing important roles in protein modification and folding, the ER as a major organelle also integrates and coordinates cellular responses to a variety of stressors (55, 56). Disturbance of the ER functions by inhibiting protein glycosylation or disrupting calcium homeostasis, oxidative stress, pathogen infection, can result in the accumulation of unfolded or misfolded proteins in the ER that will trigger the UPR. The induction of GRPs and ER-resident chaperone molecules, including GRP170, often used as an indicator of the UPR, can help overcome the excessive protein loading and maintain or recover ER functions (7, 57).

There are three major signaling pathways involved in the canonical UPR upon ER stress, which includes pancreatic ER kinase (PERK), inositol-requiring transmembrane kinase/endonuclease-1 (IRE-1), and activating transcription factor-6 (ATF6) (7, 58). These ER stress sensors upon activation will initiate a cascade of molecular events that help diminish the protein load by inhibiting protein translation or by limiting the pool of mRNAs available to enter the ER. They can concurrently engage a transcriptional program to upregulate a number of genes, e.g., ER chaperones including GRPs, X-box binding protein 1 (XBP-1), and ERAD components, which are crucial for protein folding, amino acid metabolism, and protein degradation (7, 59–61). Activated ATF6 transported to the nucleus induces the transcription of the GRPs mRNA by binding to the ER stress response element (ERSE) in their genes (58). Activated PERK phosphorylates eukaryotic initiation factor 2α (eIF2α), resulting in translation of the ATF4, which also binds to the ERSE sequence to increase the expression GRPs (58). Both ATF6 and ATF4 have been reported to mediate the UPR-dependent induction of GRP170 due to the existence of ERSE in its gene (30, 31, 62, 63). The IRE1α induces the unconventional splicing of XBP-1 mRNA and production of the longer isoform of spliced XBP-1 (XBP-1s), which stimulates the transcription of ER chaperone genes, including GRP170 (58, 64, 65).

Several lines of evidences support a cytoprotective role of intracellular GRP170 in response to ER stress. GRP170 can limit oxidized low density lipoprotein (ox-LDL)-induced ER stress and prevent subsequent cell apoptosis (66, 67). GRP170 executes this protective activity by maintaining calcium homeostasis and blocking calcium signaling through IP3 channels (67). Cytoprotection conferred by induction of GRP170 has also been shown in cellular responses to other ER stressors, e.g., proteasome inhibitors that cause excessive protein accumulation (30, 68), hypoxia, ischemia-reperfusion (69–72), and glutamate-induced cytotoxicity (73). However, the conventional UPR signaling is not the only molecular mechanism involved in the induction of GRP170. In a high fat diet-fed mouse model, AMP-activated protein kinase (AMPK) was reported to mediate the elevation of GRP170, which ameliorated hepatic ER stress and lipotoxic death. Forkhead box O1 (FOXO1), which can directly bind to the promoter region of GRP170 gene, was identified as the critical transcription factor mediating the AMPK-enhanced GRP170 expression at both the mRNA and protein levels in hepatocytes (74). However, an animal study of COX-2 inhibitor (i.e., parecoxib)-mediated neuroprotection from cerebral ischemic reperfusion injury showed that the elevation of GRP170 was observed in the presence of suppression of FOXO1 activation (75). GRP170 was found to directly bind to ER stress sensors, such as PERK, ATF6, and IRE1α in vascular cells (66) or PERK and eIF2α in hepatocytes (74), which suggests that the ER-resident GRP170 may be able to retain those ER stressors inside of ER and maintain them in an inactive state, thereby preventing the activation of ER stress.

## GRP170 in Cancer Development and Progression

Tumor development is associated with cell hyper-proliferation, protein overexpression, and emergence and accumulation of mutated or misfolded oncogenic proteins, which often induce overexpression of GRPs and other ER chaperone molecules in cancer cells (10, 15, 76). Elevation of chaperone molecules may be required for the maintenance of the functions of those proteins essential for tumorigenesis or invasion. The deprivation of glucose and hypoxic condition in the tumor microenvironment, caused by poor vascularization in most of the neoplastic tumors, can act as ER stressors that activate the UPR in cancer cells to promote their survival (77–79). It has been well documented that many ER chaperones, including GRP78, GRP94, and calreticulin (CRT), are capable of protecting cancer cells against ER stress-induced cell death (76, 80–84). Upregulation of GRP78 (85–88) and GRP94 (89–91), likely due to the adaptive UPR in cancer cells, have also been associated with the poor survival or recurrence in cancer patients as well as tumor resistance to radiotherapy.

**Figure 1:**
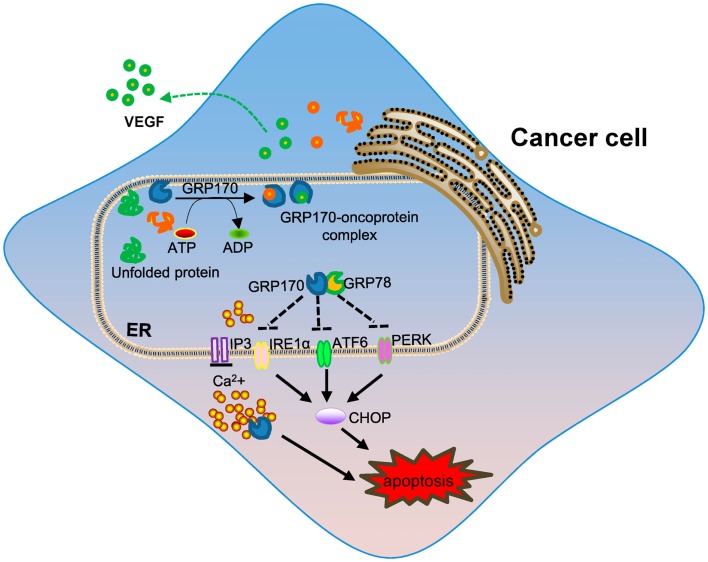
**The role of intracellular GRP170 in tumor development**. ER-resident GRP170 together with other GRPs (e.g., GRP78) or chaperone molecules participate in post-translational modification and protein folding or transportation. Under stress conditions (e.g., ER stress), GRP170 exhibits cytoprotective activity by preventing protein aggregation or help protein repair and maintaining calcium homeostasis in the ER. GRP170 can directly bind to and possibly keep the ER stress sensors (e.g., PERK, ATF6, and IRE1α) in quiescent inactivate state to limit ER stress. The pro-survival effect of intracellular GRP170 and chaperoning of oncogenic or tumor-promoting factors (e.g., VEGF) may contribute to tumor progression and invasion.

The levels of GRP170 were also shown to correlate with cancer invasiveness and GRP170 was suggested to be a potential prognostic factor in human breast cancer (92). In addition to altered expression of GRP170 in the different stage of breast cancer, the upregulation of GRP170 correlated with tumor lymph node invasion and decreased expression of estrogen receptor (93), implicating its potential involvement in cancer metastasis. Beside cytoprotection or resistance to cell death conferred by GRP170, other activities of GRP170 during tumor progression have been elucidated. Angiogenesis, formation of new capillaries from pre-existing vessels, is an important process in tumor growth and metastasis. GRP170 was shown to be required for the angiogenesis of C6 glioma tumors by facilitating the processing and secretion of vascular endothelial growth factor (VEGF), a major pro-angiogenic factor (94). Similarly, suppression of GRP170 using an antisense approach reduced the tumorigenicity of human prostate cancer cells through blocking of secretion of matured VEGF (95). GRP170 in bladder cancer cells was also found to chaperone matrix metalloproteinase-2 (MMP-2) for secretion, thereby promoting tumor invasion (96). While additional studies are needed to better understand the precise contribution of GRP170 in tumorigenesis, the chaperoning property appears to be a main underlying mechanism involved in its pro-tumor activity (Figure 1). Interestingly, it was recently found that ER-stressed tumors could propagate the stress signals to the neighboring cells (e.g., macrophages) via secretion of soluble mediators, which lead to an amplified inflammatory response that facilitates tumor progression (10, 97). It is not clear as to whether GRPs or ER chaperones in cancer cells contribute to this pro-inflammatory and pro-tumoral effect.

## Cancer Immunogenicity Altered by Compartmentalization of GRP170

Glucose-regulated proteins or ER chaperones are initially considered to be exclusively intracellular proteins that only released into extracellular environment upon cell injury (98). GRP170, like other GRPs, resides normally in the lumen of ER due to an ER-retention signal, KNDEL, at its carboxyl terminus. However, it is now apparent that ER chaperones can be present on the plasma membrane or actively secreted into the extracellular environment (10, 99–101). The differential localizations of GRPs could potentially have distinct impact on cellular activities and the host response. The cell surface GRP78 acts as a multifunctional receptor that promotes cancer cell survival and proliferation by activating ERK and AKT (102, 103), PI3k (104), or NF-κB and AKT (105). In contrast, tumor cells forced to secrete GRP78 resulted in a tumor-reactive immune response and tumor rejection (106). Another ER chaperone CRT was recently reported to translocation to the tumor cell surface upon exposure to chemotherapeutic agents or ionizing irradiation (107, 108). The surface CRT serves as an “eat me” signal that triggers increased phagocytosis of dying tumor cells, cross-presentation of tumor antigens, and consequent anti-tumor immune response (109).

We have performed studies to determine the impact of extracellular secretion of GRP170 on tumorigenicity. In this regard, murine B16 melanoma cells (47), TRAMP-C2 prostate cancer cells (110), or CT26 colorectal cancer cells (111) were forced to express a secretable form of GRP170, in which its ER-retention sequence “KNDEL” has been depleted. We found that these cancer cells secreting GRP170 did not differ from their mock-treated controls in cell proliferation *in vitro*. However, the tumor growth was markedly suppressed *in vivo*, which was dependent on the presence of cytotoxic CD8^+^ T lymphocytes (CTLs) and/or natural killer (NK) cells. This secretory GRP170 not only acted as a “danger” signal stimulating specialized antigen-presenting cells (APCs), such as dendritic cells (DCs), but also delivered tumor-derived antigens via its intrinsic chaperoning activity for priming antigen-specific CTLs (47). Using mass spectrometry analysis, we demonstrated that this secreted GRP170 was associated with tumor protein antigens (111), which is consistent with the intracellular chaperoning function of GRPs or ER chaperones that are essential for the activity of oncoproteins in cancer cells.

The studies of cell surface GRP94 (112) or GRP94 secreted from tumor cells (113–115) support our findings by demonstrating that the exposure of GRP94 to the immune system represents a highly immunogenic signal capable of inducing a potent anti-tumor immune response. Further support came from a study showing that enforced cell surface expression of GRP94 in a transgenic mouse conferred hyper-responsiveness to LPS and induced lupus-like autoimmune syndrome (116, 117). Thus, GRP170 or other ER chaperones may display dual biological or immunological activities during tumor progression or in response to therapeutic treatments, which depends on individual GRPs as well as their cellular compartmentalization. Intracellular or ER-resident GRPs play a general protective role that promotes the survival of cancer cells in stress or lethal conditions. However, the membrane-bound or extracellular GRPs could alter the immunogenicity of cancer cells and facilitate the immune recognition as well as immune-mediated destruction of cancers (10, 15, 16, 118–120). Manipulating cellular compartmentalization of GRP170 may be used to induce or restore protective anti-tumor immunity for cancer eradication. Genetically modified cancer cells with a capacity to produce secretory GRP170 have been successfully tested as a cell-based vaccine to generate a therapeutic anti-tumor response to the established tumors in mice (47, 110). We also demonstrated that intratumoral delivery of secretory GRP170 via an adenovirus promoted the anti-tumor efficacy of melanoma differentiation-associated gene-7 (mda-7), a cancer-specific therapeutic cytokine, by mounting systemic anti-tumor immunity that controls treated as well as distant untreated tumor lesions (121).

## GRP170 as an Immune Adjuvant of Cancer Vaccine

Glucose-regulated protein preparations or ER chaperones-derived from tumors are believed to carry individually distinct array of tumor antigens, which can be utilized to provoke a tumor-specific immune response in cancer vaccination or immunotherapy (25, 122–128). Our early studies showed that animals immunized with GRP170 purified from various murine tumors (e.g., colon tumor, melanoma, and fibrosarcoma) developed a robust anti-tumor immune response (126, 129). Tumor-derived GRP170, when compared to other chaperones, displayed a higher anti-tumor potency (129), which, we believe, is attributed to its superior protein or antigen-holding capacity (48). The substantially increased size of GRP170 due to the extension of its C-terminal domain could be a factor, which enables it to bind to and chaperone protein clients or antigens more efficiently (14, 16). Indeed, two independent regions in GRP170, i.e., the classical peptide-binding β-sheet domain and the C-terminal α-helix domain, have been identified that can execute chaperoning activities (37).

To overcome the hurdle of preparing autologous GRP vaccines for clinical use, which can be limited by a requirement of patient specimen and laborious procedures of vaccine production (130, 131), we have developed a recombinant chaperoning technology that exploits the exceptional antigen-holding capacity of GRP170 (132). The reconstituted chaperone complex of GRP170 and melanoma-associated antigen gp100 has been used as a targeted vaccine to generate a strong anti-tumor immune response to aggressive, poorly immunogenic B16 melanoma in mice (132). Similar observations have been made in other vaccination studies that employed GRP170 to target different tumor antigens (133, 134). This recombinant vaccine approach has several advantages over autologous vaccines. The GRP170-antigen complex vaccine can be prepared in large quantities for use off-the-shelf. A large reservoir of antigenic epitopes in a protein antigen can stimulate polyepitope-directed T cells and potentially enhance the strength of the anti-tumor effect. Our recent comparison study of GRP170-protein antigen complex vs. GRP170-peptide antigen in the setting of therapeutic immunization against cancer strongly supports the idea of including protein antigen in this vaccine regimen (48). Use of defined antigenic target should also facilitate the monitoring of antigen-specific immune responses in the clinic. Additionally, the highly efficient antigen-holding property will permit the development of a multivalent vaccine against different antigenic targets (48).

## Extracellular GRP170 and Antigen Cross-Presentation

Dendritic cells are one of most efficient APCs for processing and presenting antigens to T lymphocytes. Cross-priming of the CD8^+^ T cells by DCs plays a crucial role in the induction of antiviral and anti-tumor immune responses. The cross-presentation efficacy of DCs is determined by many factors, including their ability to capture antigens, the route of antigen uptake and trafficking, antigen stability, and the pathways by which processed antigen is loaded on the MHC class I molecules. Generally, two models have been proposed for antigen cross-presentation, i.e., the vacuolar pathway and the cytosolic pathway (135, 136). In the vacuolar pathway, internalized antigens remain in endolysosomal/phagosomal compartments, where they are degraded and loaded onto the recycling MHC class I molecules (137–139). In the cytosolic pathway, the endocytosed antigens are transported from endosomes or phagosomes into the cytosol for proteasome-dependent degradation, followed by peptide import and loading onto MHC class I molecules in the ER (140–143).

The choice of adjuvant is critical in the success of antigen-targeted, protein-based cancer vaccines because soluble protein antigens are typically poorly cross-presented by DCs. One of major tenets in the vaccine activity of GRPs and other chaperone molecules, including GRP170, is their high efficiency to introduce antigens into the endogenous antigen-processing pathway of APCs for cross-presentation and activation of CTLs (48, 132, 144–147). The interaction of intracellular GRP170 with TAP in the early studies suggested that endogenous GRP170 may assist with ER translocation of peptides (41, 42). Two scavenger receptors, SR-A and SREC-I, have been identified to contribute to the binding of GRP170 on APCs (148). However, the mechanism of cross-presentation enhanced by exogenously delivered GRP170 in the context of tumor vaccination is poorly defined.

Using a clinically relevant melanoma antigen gp100 carried by GRP170, we recently investigated the trafficking pathways of GRP170–gp100 complex in DCs. Surprisingly, we found that the GRP170 directed and enhanced gp100 efficiently to access the ER after their internalization. GRP170-facilitated gp100 processing and presentation was dependent on the ERAD machinery involving Sec61, which was shown to target gp100 for ubiquitination and degradation in the cytosol by the proteasome system and subsequent integration into the conventional MHC class I restricted antigen-processing pathway (149). Our data indicated that GRP170 can help the associated protein antigen escape from lysosomal degradation and shuttle the antigen into the ER compartment from the early endosomal compartment. Internalized GRP170 might be directly involved in the ERAD following vaccine uptake, because GRP170 in the complex enhanced the interaction of gp100 with several ERAD molecules (e.g., Sec61α, VCP/97, CHIP, and GRP78). We speculate that gp100 protein that is partially unfolded during the vaccine preparation and chaperoned by GRP170 serves as an ERAD target once accessing the ER. Since endogenous GRP170 also binds to Sec61α, it is likely that internalized GRP170 could become a part of the ER chaperone network and collaborate with other GRPs to guide retrotranslocation of gp100. The GRP170/Lhs in yeast was recently reported to facilitate the ERAD of the epithelial sodium channel by preferentially targeting the unglycosylated form of the protein, which relied on its holding function not NEF activity (150). In our studies, the transient co-localization of ER markers with early endosome marker suggested an interesting possibility of formation of ER/endosome fusion structure after vaccine captured by DCs, which may explain the route of the ER access of GRP170–gp100 complex from the extracellular environment. Intriguingly, the GRP170-peptide antigen complex was recently found to be transported into early and recycling endosome compartments, where antigen was processed (151). It appears that the distinct trafficking patterns are caused by the size or nature of the antigens (protein vs. peptide) chaperoned by GRP170 in vaccines.

ER-associated degradation is an essential protein quality control mechanism in the ER that retrotranslocates unfolded or misfolded proteins to cytosol for degradation in response to ER stress (152, 153). ERAD involving Sec61 and chaperone molecules have been implicated in the cytosolic pathway of antigen cross-presentation (137, 154). Several lines of evidence suggest that the ERAD components are present on or can be recruited to the endosome/phagosome in APCs to facilitate cytosolic translocation of antigen (155–158). However, ER access and ERAD-mediated processing of GRP170–antigen complex in the setting of therapeutic vaccination warrant more studies, which will result in a better understanding of the action of this molecular adjuvant and the optimization of GRP170-based targeted cancer vaccination strategies.

## Extracellular GRP170 as an Alarmin and Innate Immunity

Upon release from injured or stressed cells, certain chaperone molecules, including GRPs, are suggested to serve as alarmins or damage-associated molecular patterns to alert the host immune systems of cell or tissue stress and trauma (159, 160). It has been well established that GRP170-dependent tumor immunization offers effective treatment of malignancies in the experimental models. The acquired immunity enhanced by GRP170 through shuttling and presenting tumor antigens for T cell cross-priming is an essential component of this process. The previous studies showed that GRP170 bound to DCs in a receptor-mediated fashion (i.e., scavenger receptors SR-A and SREC-I) (148) and GRP170 by itself could modestly induce DCs to upregulate MHC class II and co-stimulatory molecules (e.g., CD86) (120). Binding of GRP170 to DCs also stimulated them to produce pro-inflammatory cytokines (120). Although this stimulatory effect appears to be modest, it distinguishes GRP170 or other GRPs from other conventional adjuvants in vaccine design and formulation.

Vaccine adjuvants can be functionally divided into two major groups, toll-like receptor (TLR)-dependent and TLR-independent adjuvants (161, 162). TLR-dependent adjuvants, such as the Bacillus Calmette–Guerin (BCG) that is recognized by TLR2 and TLR4, act directly on DCs and promote their maturation and migration to the T cell area of the lymph node (161, 162). TLR-independent adjuvants, e.g., alum, increase antigen availability at injection site by adsorption and entrapment of antigens (163, 164). GRP170 along with other chaperone molecules, as self-proteins of mammalian origin, may be considered as the third functional group of adjuvants, because they increase the immunogenicity of antigens via preferentially delivering antigen cargos to DCs and enhancing antigen cross-presentation by DCs (120, 132, 148, 149, 165).

In addition to chaperoning intracellular antigenic polypeptides, GRP170 can efficiently bind to foreign pathogen-associated molecular patterns (PAMPs) in the extracellular environment and enhance the host response to pathogens. We recently showed that GRP170 interacts with microbial DNA, e.g., CpG oligodeoxynucleotides (CpG-ODN), a ligand for TLR9 (166). Chaperoning of CpG-ODN by extracellular GRP170 resulted in markedly increased internalization of CpG-ODN by macrophages. The internalized GRP170 was seen to directly associate with endosomal TLR9, suggesting that GRP170 chaperoning may enhance the sensing of CpG-ODN by its receptor (i.e., TLR9). As a result, complexing of CpG-ODN with GRP170 leads to enhanced activation of the MyD88-dependent signaling cascade and production of pro-inflammatory cytokines for pathogen clearance (166). Indeed, GRP170-amplified innate immune response protected mice from challenge with *Listeria monocytogenes* (166). These results revealed a previously unrecognized attribute of GRP170 as a superior DNA-binding chaperone. More importantly, the interaction of an evolutionarily conserved chaperone molecule with PAMPs in the extracellular milieu may play a critical role in the host response to pathogen. Interestingly, other than internalized GRP170, TLR9 was associated with major endogenous GRPs, including GRP170, GRP94, and GRP78 (166), suggesting that the outside-in GRP170 may function in concert with intracellular chaperone networks in modifying TLR9 signaling. This result, together with a recent work showing a critical requirement of the chaperoning of TLR9 by intracellular GRP94 for TLR9 functions (167), offers new insight into the dynamics of ancient chaperoning functions inside and outside the cell. Given that CpG-ODN can be used as an immunostimulatory adjuvant in cancer vaccination (168), the unique characteristics of GRP170 in amplifying CpG-ODN-induced immune activation provide a scientific rationale for including the CpG-ODN as a component in the recombinant GRP170 vaccine regimen for cancer immunotherapy.

**Figure 2:**
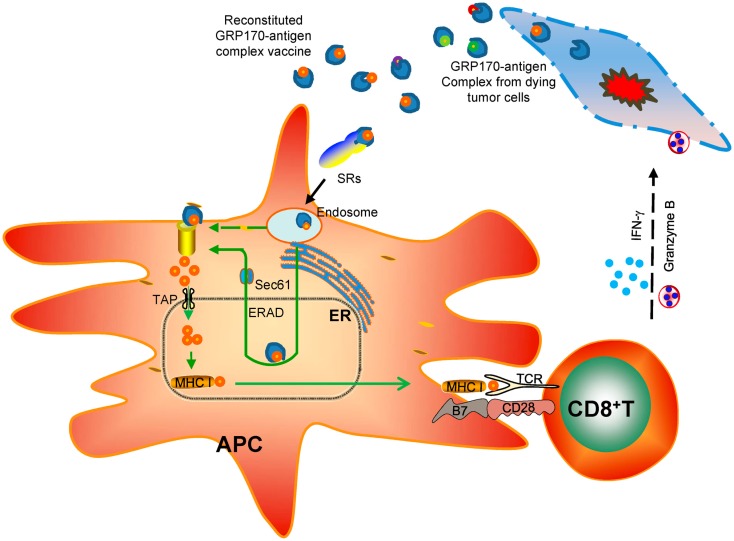
**Chaperoning-based immunological effects of extracellular GRP170 in cancer therapy**. GRP170 isolated or released from cancer cells due to stress or injury is believed to chaperone tumor antigens. These tumor-derived GRP170-antigen complexes in the extracellular environment or reconstituted recombinant GRP170-antigen complex vaccine can be captured preferentially by specialized antigen-presenting cells (APCs) through the surface receptors (e.g., scavenger receptors, SRs). The GRP170 can direct the chaperone complexes to the endoplasmic reticulum (ER) and facilitate their interaction with the components of ER-associated degradation (ERAD) machinery (e.g., Sec61α). The antigen target will then be retrotranslocated to the cytosol for ubiquitination and proteasome-mediated processing. The generated antigenic peptides are transported by TAP and loaded on the MHC class I molecules. The MHC I-peptide cargo will traffick to the cell surface and prime CD8^+^ T cells. Activation and expansion of antigen-specific CD8^+^ T cells leads to eradication of antigen-positive tumor cells by releasing cytotoxic molecules (e.g., IFN-γ, granzyme B).

Among all the biological and immunological activities of extra- cellular GRP170, e.g., enhanced endocytosis of protein antigen or CpG-ODN, increased ER access of protein antigen, increased association with TLR9, all these processes seem to intimately involve the intrinsic chaperoning property of GRP170. During investigation of vaccine potential of various deletion mutant of GRP170 (37), we found that only chaperoning competent mutants exhibited APC binding activities and could deliver tumor antigen (e.g., gp100) for inducing an antigen-specific anti-tumor immunity (132). Interestingly, two of chaperoning competent GRP170 mutants, although both contained no overlapping sequences, could still bind to APCs in a receptor-mediated fashion and stimulate tumor-inhibiting CTL response. Together, these findings support the notion that the ancient chaperoning property is the key denominator underlying the diverse biological and immunological effects of GRP170 and possibly those other immunostimulatory GRPs (Figure 2).

## Arming GRP170 with a Pathogen-Derived “Danger” Signal for Improved Anti-Tumor Potency

Coupling antigen and an immunostimulating “danger” signal into the same vaccine delivery cargo is crucial for optimal antigen cross-presentation by DCs and priming of antigen-reactive T cells (169, 170). While certain chaperone molecules in the extracellular environment, including GRP170, possess direct immunostimulatory activity during interaction with APCs, they do not activate an innate immune response as efficiently or robustly as PAMPs, which strongly promote a vaccine response (171, 172). The modest innate-stimulating effect of GRP170 may not be sufficient to fully activate antigen-exposed APCs *in vivo*. We hypothesized that incorporating a pathogen-derived “danger” signal into the GRP170 backbone would enhance its immunostimulatory potency in therapeutic immunization against cancer. To test this concept, we engineered a chimeric chaperone, termed Flagrp170, by fusing GRP170 with the defined NF-κB-activating domain of Flagellin (173).

Flagellin is the principal substituent of bacterial flagella and the ligand for TLR5 (174–176). Since the NF-κB pathways in DCs are essential for its optimal functions (169, 177–179), this chimeric chaperone possesses two distinct features that are required for efficient cancer vaccine therapy: enhancing the cross-presentation of tumor antigens in the chaperone complex cargo and concurrently provoking the functional activation of DCs via engaging the NF-κB signaling (173). As expected, Flagrp170 strongly activated DCs, indicated by elevation of co-stimulatory molecules, such as CD40 and CD86, as well as production of pro-inflammatory and Th1-polarizing cytokine IL-12. Since only a small portion of Flagellin was present in the construct, it was surprising that Flagrp170 exhibited a similar effect as Flagellin in stimulating NF-κB and MAPK signaling, as well as phenotypic activation of DCs. This might be due to the ability of GRP170 to amplify the innate immune response, as we observed in the study of GRP170 interaction with CpG-ODN (166). Moreover, Flagrp170 was much more efficacious than Flagellin in promoting antigen cross-presentation, which can be explained by the superior intrinsic property of GRP170 in antigen shuttling and T cell cross-priming (149).

Intratumoral delivery of Flagrp170 using an adenovirus induced a superior anti-tumor response against treated B16 melanoma and distant lung metastases compared with unmodified GRP170 or Flagellin treatment (173), which indicates systemic mobilization of tumor-reactive immune effector cells. Flagrp170 treatment was shown to drive Th1 polarization of the tumor microenvironment, characterized by high levels of IL-12 and IFN-γ, as well as tumor-infiltrating CD8^+^ and NK cells (173). Mechanistic studies showed that depletion of CD11c^+^ cells or lack of CD8α^+^ DCs attenuated the anti-tumor response generated by Flagrp170 therapy, suggesting that Flagrp170-enhanced activation of tumor-specific CTLs depends on these DCs for efficient antigen cross-presentation (173). Interestingly, Flagrp170 selectively activated the NF-κB signaling pathway in DCs, not in tumor cells, which suggests that Flagrp170 represents an ideal agent that may be exploited to condition the immunosuppressive tumor environment and to break immune tolerance established during tumor development or progression.

## Conclusion

Glucose-regulated protein 170, as one of the largest GRPs and chaperone molecules in the ER, can protect cells during ER stress-triggered UPR and in other stressful and lethal conditions. The cytoprotective activity of GRP170 is also reflected in its elevation in certain cancer cells and resultant resistance of tumor cells to the induction of cell death, which supports a potential tumor-promoting role of GRP170 during cancer progression. While the intracellular chaperoning and NEF functions of GRP170 remain to be further defined, accumulating evidence has highlighted an immunoregulatory effect of GRP170 in the extracellular environment, indicated by its superior capacity in holding protein tumor antigens, facilitating antigen cross-presentation, enhancing T cell priming, and amplifying an innate immune response. These unique features have been exploited to develop GRP170 chaperone complex vaccine directed against defined antigenic target in cancers. The successful results derived from preclinical models have led to an ongoing phase I clinical trial of testing recombinant chaperone vaccine in melanoma patients. While this vaccine approach holds promise, more studies are needed to better understand the adjuvant action of GRP170 in therapeutic immunization. Studies are also needed with regard to the contributions of other DC subsets in GRP170-enhanced antigen cross-presentation, such as lymphoid organ resident CD8α^+^ DCs and dermal migratory CD103^+^ DCs (180–183). Recently, the role of the IRE1α–XBP-1 pathway has been extended beyond UPR and was shown to be required for the differentiation of effector CD8^+^ T cells and development or function of DCs (184–186). It is of interest to explore the potential involvement of GRP170 and other GRPs in this context because both intracellular and extracellular GRPs are likely to actively participate in these processes given their documented roles in antigen transportation, processing, and presentation. Integrating a NF-κB-stimulating “danger” signal into the GRP170-based delivery cargo strongly enhances its immunostimulatory and anti-tumor efficacy, which warrants the future studies of Flagrp170 as a novel immunomodulating agent either alone or combined with conventional treatment modalities (chemotherapy and radiotherapy) to restore anti-tumor immunity in the tumor site to achieve *in situ* vaccination. It is also conceivable that this engineered GRP170 molecule can be used to design the new generation of targeted chaperone vaccine to deliver tumor protein antigens for the treatment of metastatic malignancies.

## Conflict of Interest Statement

The authors declare that the research was conducted in the absence of any commercial or financial relationships that could be construed as a potential conflict of interest.
